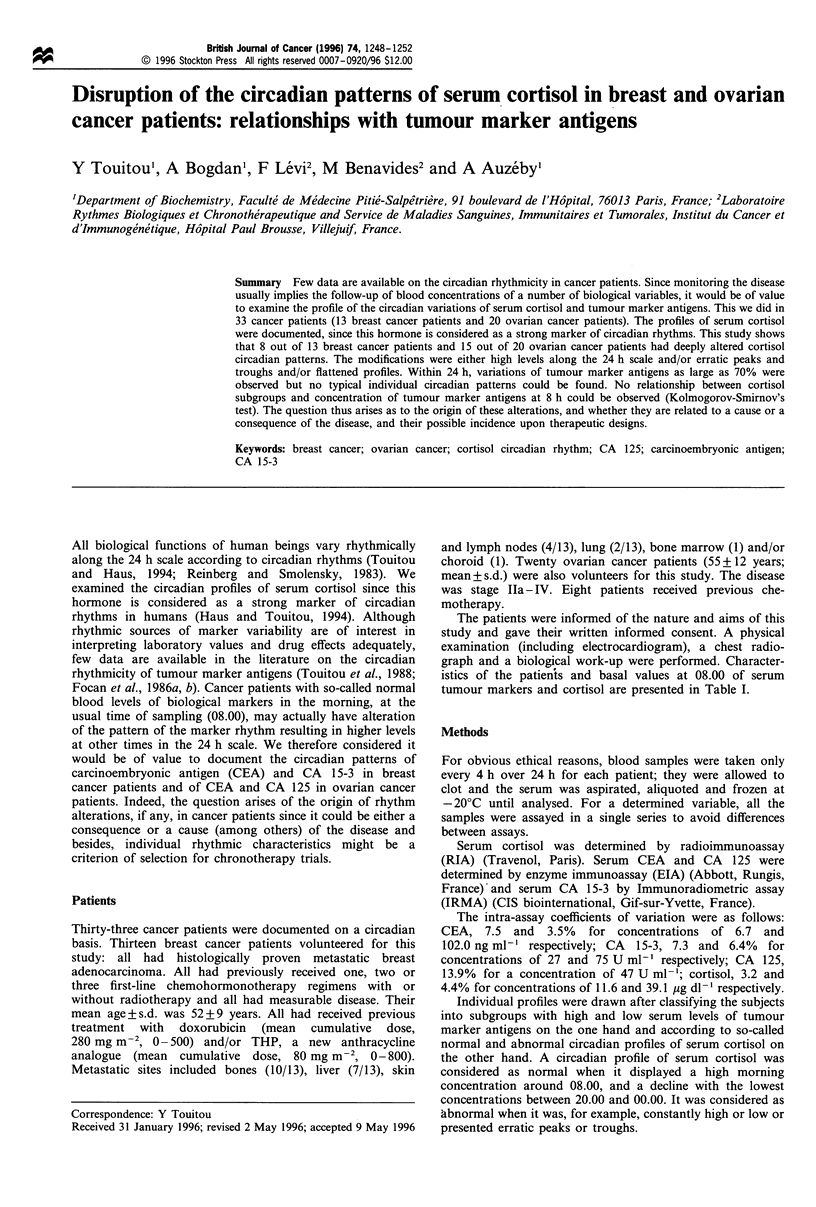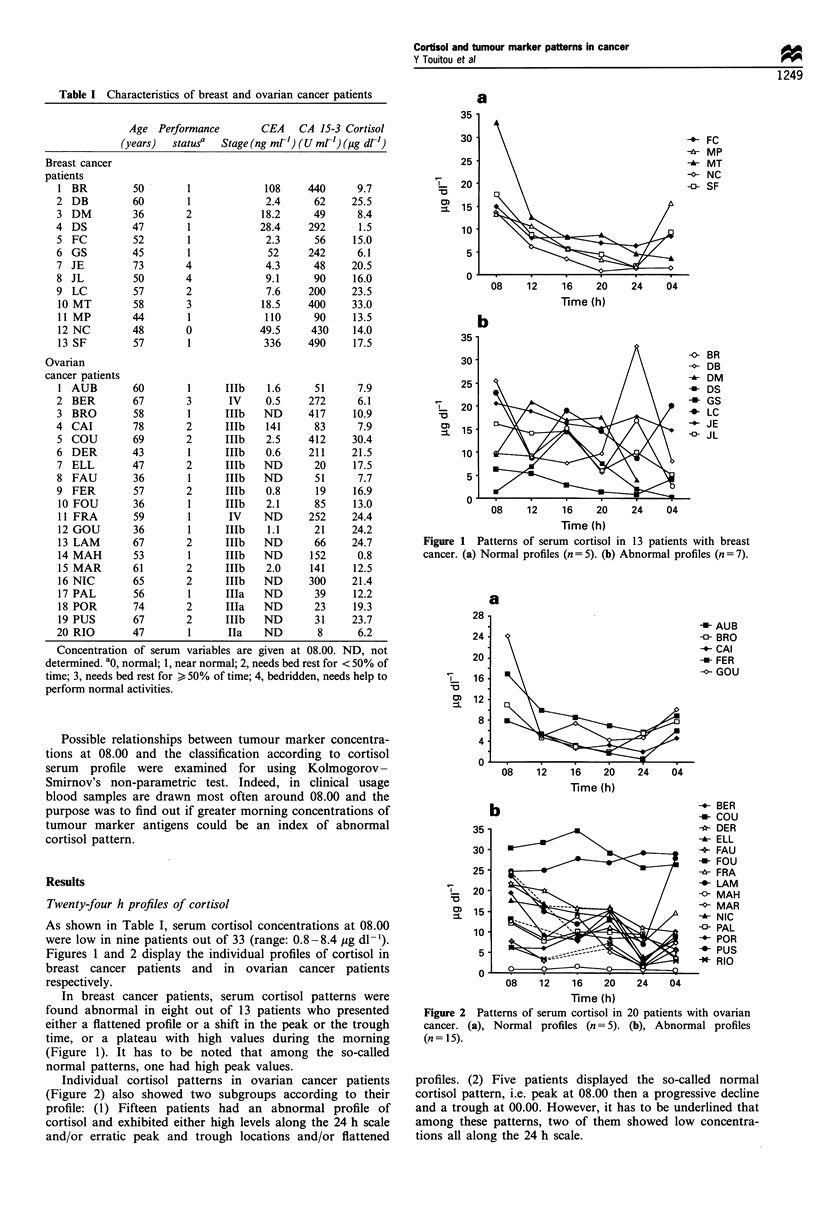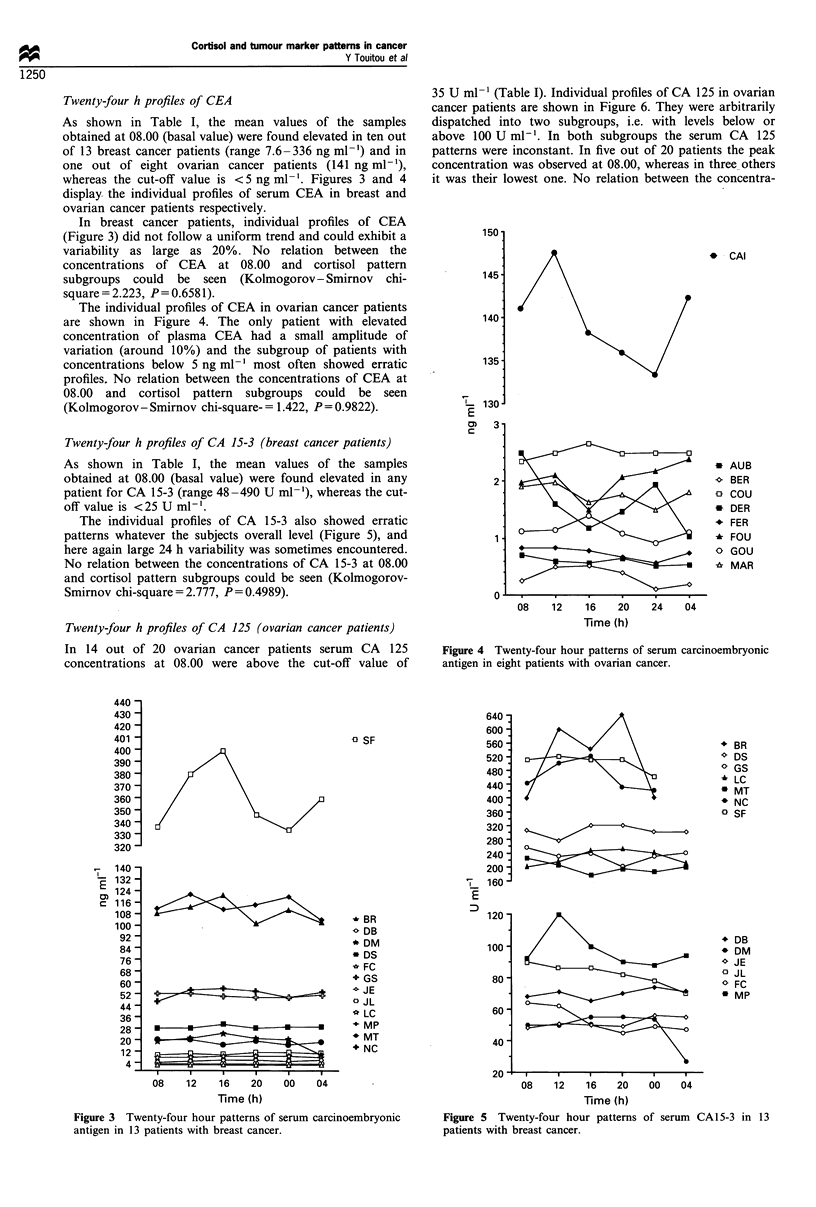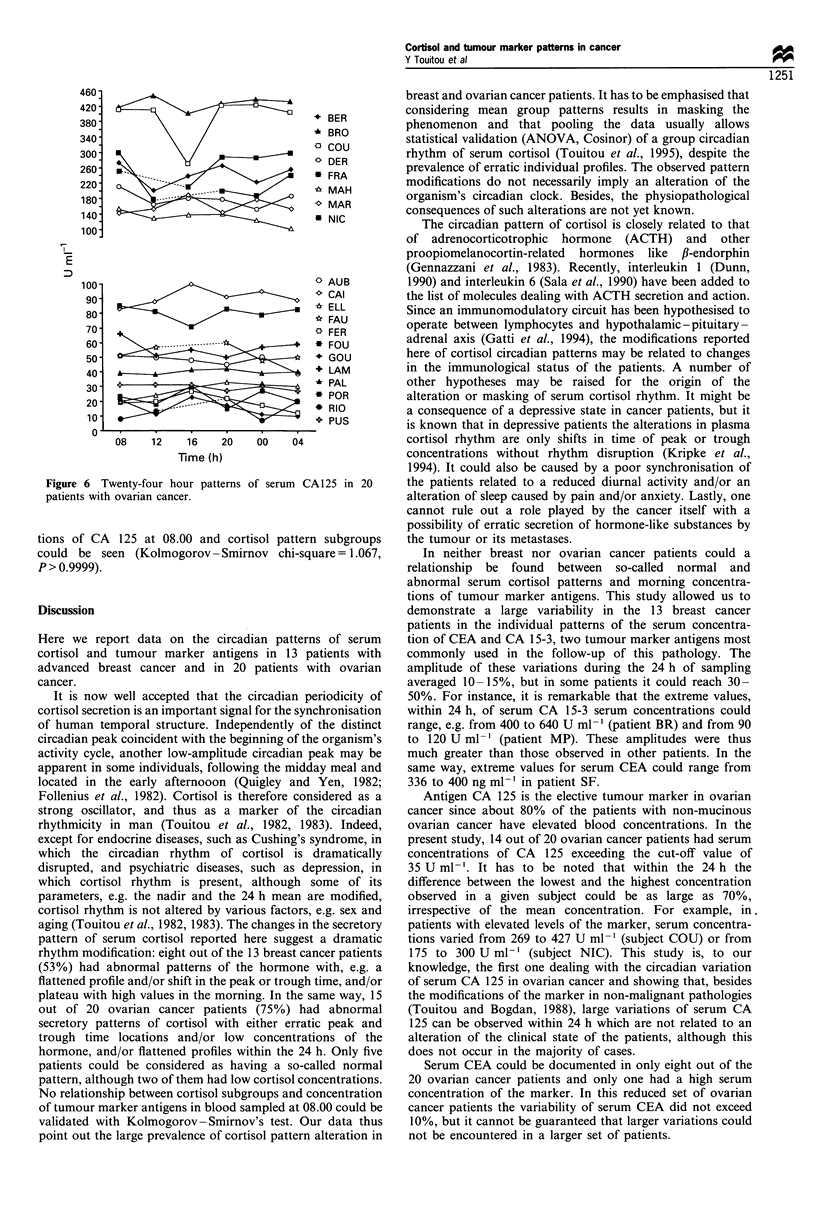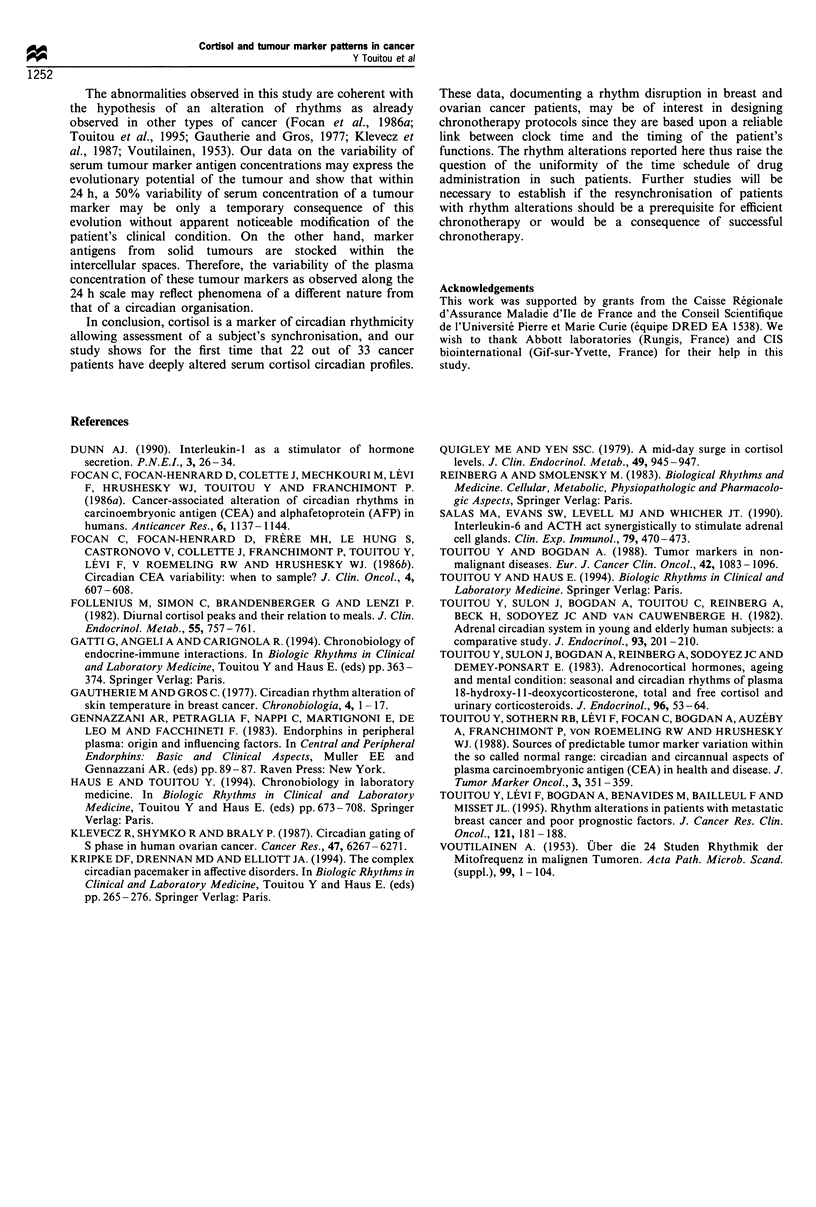# Disruption of the circadian patterns of serum cortisol in breast and ovarian cancer patients: relationships with tumour marker antigens.

**DOI:** 10.1038/bjc.1996.524

**Published:** 1996-10

**Authors:** Y. Touitou, A. Bogdan, F. Lévi, M. Benavides, A. Auzéby

**Affiliations:** Department of Biochemistry, Faculté de Médecine Pitié-Salpêtrière, Paris, France.

## Abstract

Few data are available on the circadian rhythmicity in cancer patients. Since monitoring the disease usually implies the follow-up of blood concentrations of a number of biological variables, it would be of value to examine the profile of the circadian variations of serum cortisol and tumour marker antigens. This we did in 33 cancer patients (13 breast cancer patients and 20 ovarian cancer patients). The profiles of serum cortisol were documented, since this hormone is considered as a strong marker of circadian rhythms. This study shows that 8 out of 13 breast cancer patients and 15 out of 20 ovarian cancer patients had deeply altered cortisol circadian patterns. The modifications were either high levels along the 24 h scale and/or erratic peaks and troughs and/or flattened profiles. Within 24 h, variations of tumour marker antigens as large as 70% were observed but no typical individual circadian patterns could be found. No relationship between cortisol subgroups and concentration of tumour marker antigens at 8 h could be observed (Kolmogorov-Smirnov's test). The question thus arises as to the origin of these alterations, and whether they are related to a cause or a consequence of the disease, and their possible incidence upon therapeutic designs.


British Journal of Cancer (1996) 74, 1248-1252
© 1996 Stockton Press All rights reserved 0007-0920/96 $12.00

### Disruption of the circadian patterns of serum cortisol in breast and ovarian cancer patients: relationships with tumour marker antigens

Y Touitou[1], A Bogdan[1], F Lévi[2], M Benavides[2] and A Auzéby[1]

[1]*Department of Biochemistry, Faculté de Médecine Pitié-Salpêtrière, 91 boulevard de l'Hôpital, 76013 Paris, France;* [2]*Laboratoire Rythmes Biologiques et Chronothérapeutique and Service de Maladies Sanguines, Immunitaires et Tumorales, Institut du Cancer et d'Immunogénétique, Hôpital Paul Brousse, Villejuif, France.*

**Summary** Few data are available on the circadian rhythmicity in cancer patients. Since monitoring the disease usually implies the follow-up of blood concentrations of a number of biological variables, it would be of value to examine the profile of the circadian variations of serum cortisol and tumour marker antigens. This we did in 33 cancer patients (13 breast cancer patients and 20 ovarian cancer patients). The profiles of serum cortisol were documented, since this hormone is considered as a strong marker of circadian rhythms. This study shows that 8 out of 13 breast cancer patients and 15 out of 20 ovarian cancer patients had deeply altered cortisol circadian patterns. The modifications were either high levels along the 24 h scale and/or erratic peaks and troughs and/or flattened profiles. Within 24 h, variations of tumour marker antigens as large as 70% were observed but no typical individual circadian patterns could be found. No relationship between cortisol subgroups and concentration of tumour marker antigens at 8 h could be observed (Kolmogorov-Smirnov's test). The question thus arises as to the origin of these alterations, and whether they are related to a cause or a consequence of the disease, and their possible incidence upon therapeutic designs.

**Keywords:** breast cancer; ovarian cancer; cortisol circadian rhythm; CA 125; carcinoembryonic antigen; CA 15-3

All biological functions of human beings vary rhythmically along the 24 h scale according to circadian rhythms (Touitou and Haus, 1994; Reinberg and Smolensky, 1983). We examined the circadian profiles of serum cortisol since this hormone is considered as a strong marker of circadian rhythms in humans (Haus and Touitou, 1994). Although rhythmic sources of marker variability are of interest in interpreting laboratory values and drug effects adequately, few data are available in the literature on the circadian rhythmicity of tumour marker antigens (Touitou *et al.*, 1988; Focan *et al.*, 1986*a*, *b*). Cancer patients with so-called normal blood levels of biological markers in the morning, at the usual time of sampling (08.00), may actually have alteration of the pattern of the marker rhythm resulting in higher levels at other times in the 24 h scale. We therefore considered it would be of value to document the circadian patterns of carcinoembryonic antigen (CEA) and CA 15-3 in breast cancer patients and of CEA and CA 125 in ovarian cancer patients. Indeed, the question arises of the origin of rhythm alterations, if any, in cancer patients since it could be either a consequence or a cause (among others) of the disease and besides, individual rhythmic characteristics might be a criterion of selection for chronotherapy trials.

### Patients

Thirty-three cancer patients were documented on a circadian basis. Thirteen breast cancer patients volunteered for this study: all had histologically proven metastatic breast adenocarcinoma. All had previously received one, two or three first-line chemohormonotherapy regimens with or without radiotherapy and all had measurable disease. Their mean age ± s.d. was 52 ± 9 years. All had received previous treatment with doxorubicin (mean cumulative dose, 280 mg $m^{-2}$, 0-500) and/or THP, a new anthracycline analogue (mean cumulative dose, 80 mg $m^{-2}$, 0-800). Metastatic sites included bones (10/13), liver (7/13), skin and lymph nodes (4/13), lung (2/13), bone marrow (1) and/or choroid (1). Twenty ovarian cancer patients (55 ± 12 years; mean ± s.d.) were also volunteers for this study. The disease was stage IIa-IV. Eight patients received previous chemotherapy.

The patients were informed of the nature and aims of this study and gave their written informed consent. A physical examination (including electrocardiogram), a chest radiograph and a biological work-up were performed. Characteristics of the patients and basal values at 08.00 of serum tumour markers and cortisol are presented in Table I.

### Methods

For obvious ethical reasons, blood samples were taken only every 4 h over 24 h for each patient; they were allowed to clot and the serum was aspirated, aliquoted and frozen at −20°C until analysed. For a determined variable, all the samples were assayed in a single series to avoid differences between assays.

Serum cortisol was determined by radioimmunoassay (RIA) (Travenol, Paris). Serum CEA and CA 125 were determined by enzyme immunoassay (EIA) (Abbott, Rungis, France) and serum CA 15-3 by Immunoradiometric assay (IRMA) (CIS biointernational, Gif-sur-Yvette, France).

The intra-assay coefficients of variation were as follows: CEA, 7.5 and 3.5% for concentrations of 6.7 and 102.0 ng $ml^{-1}$ respectively; CA 15-3, 7.3 and 6.4% for concentrations of 27 and 75 U $ml^{-1}$ respectively; CA 125, 13.9% for a concentration of 47 U $ml^{-1}$; cortisol, 3.2 and 4.4% for concentrations of 11.6 and 39.1 $\mu g$ $dl^{-1}$ respectively.

Individual profiles were drawn after classifying the subjects into subgroups with high and low serum levels of tumour marker antigens on the one hand and according to so-called normal and abnormal circadian profiles of serum cortisol on the other hand. A circadian profile of serum cortisol was considered as normal when it displayed a high morning concentration around 08.00, and a decline with the lowest concentrations between 20.00 and 00.00. It was considered as abnormal when it was, for example, constantly high or low or presented erratic peaks or troughs.

Correspondence: Y Touitou
Received 31 January 1996; revised 2 May 1996; accepted 9 May 1996

**Table I** Characteristics of breast and ovarian cancer patients

| | *Age (years)* | *Performance status*[a] | *Stage* | *CEA (ng ml$^{-1}$)* | *CA 15-3 (U ml$^{-1}$)* | *Cortisol (µg dl$^{-1}$)* |
|---|---|---|---|---|---|---|
| Breast cancer patients | | | | | | |
| 1 BR | 50 | 1 | | 108 | 440 | 9.7 |
| 2 DB | 60 | 1 | | 2.4 | 62 | 25.5 |
| 3 DM | 36 | 2 | | 18.2 | 49 | 8.4 |
| 4 DS | 47 | 1 | | 28.4 | 292 | 1.5 |
| 5 FC | 52 | 1 | | 2.3 | 56 | 15.0 |
| 6 GS | 45 | 1 | | 52 | 242 | 6.1 |
| 7 JE | 73 | 4 | | 4.3 | 48 | 20.5 |
| 8 JL | 50 | 4 | | 9.1 | 90 | 16.0 |
| 9 LC | 57 | 2 | | 7.6 | 200 | 23.5 |
| 10 MT | 58 | 3 | | 18.5 | 400 | 33.0 |
| 11 MP | 44 | 1 | | 110 | 90 | 13.5 |
| 12 NC | 48 | 0 | | 49.5 | 430 | 14.0 |
| 13 SF | 57 | 1 | | 336 | 490 | 17.5 |
| Ovarian cancer patients | | | | | | |
| 1 AUB | 60 | 1 | IIIb | 1.6 | 51 | 7.9 |
| 2 BER | 67 | 3 | IV | 0.5 | 272 | 6.1 |
| 3 BRO | 58 | 1 | IIIb | ND | 417 | 10.9 |
| 4 CAI | 78 | 2 | IIIb | 141 | 83 | 7.9 |
| 5 COU | 69 | 2 | IIIb | 2.5 | 412 | 30.4 |
| 6 DER | 43 | 1 | IIIb | 0.6 | 211 | 21.5 |
| 7 ELL | 47 | 2 | IIIb | ND | 20 | 17.5 |
| 8 FAU | 36 | 1 | IIIb | ND | 51 | 7.7 |
| 9 FER | 57 | 2 | IIIb | 0.8 | 19 | 16.9 |
| 10 FOU | 36 | 1 | IIIb | 2.1 | 85 | 13.0 |
| 11 FRA | 59 | 1 | IV | ND | 252 | 24.4 |
| 12 GOU | 36 | 1 | IIIb | 1.1 | 21 | 24.2 |
| 13 LAM | 67 | 2 | IIIb | ND | 66 | 24.7 |
| 14 MAH | 53 | 1 | IIIb | ND | 152 | 0.8 |
| 15 MAR | 61 | 2 | IIIb | 2.0 | 141 | 12.5 |
| 16 NIC | 65 | 2 | IIIb | ND | 300 | 21.4 |
| 17 PAL | 56 | 1 | IIIa | ND | 39 | 12.2 |
| 18 POR | 74 | 2 | IIIa | ND | 23 | 19.3 |
| 19 PUS | 67 | 2 | IIIb | ND | 31 | 23.7 |
| 20 RIO | 47 | 1 | IIa | ND | 8 | 6.2 |

Concentration of serum variables are given at 08.00. ND, not determined. [a]0, normal; 1, near normal; 2, needs bed rest for <50% of time; 3, needs bed rest for ≥50% of time; 4, bedridden, needs help to perform normal activities.

Possible relationships between tumour marker concentrations at 08.00 and the classification according to cortisol serum profile were examined for using Kolmogorov–Smirnov's non-parametric test. Indeed, in clinical usage blood samples are drawn most often around 08.00 and the purpose was to find out if greater morning concentrations of tumour marker antigens could be an index of abnormal cortisol pattern.

### Results

### *Twenty-four h profiles of cortisol*

As shown in Table I, serum cortisol concentrations at 08.00 were low in nine patients out of 33 (range: 0.8–8.4 µg dl$^{-1}$). Figures 1 and 2 display the individual profiles of cortisol in breast cancer patients and in ovarian cancer patients respectively.

In breast cancer patients, serum cortisol patterns were found abnormal in eight out of 13 patients who presented either a flattened profile or a shift in the peak or the trough time, or a plateau with high values during the morning (Figure 1). It has to be noted that among the so-called normal patterns, one had high peak values.

Individual cortisol patterns in ovarian cancer patients (Figure 2) also showed two subgroups according to their profile: (1) Fifteen patients had an abnormal profile of cortisol and exhibited either high levels along the 24 h scale and/or erratic peak and trough locations and/or flattened profiles. (2) Five patients displayed the so-called normal cortisol pattern, i.e. peak at 08.00 then a progressive decline and a trough at 00.00. However, it has to be underlined that among these patterns, two of them showed low concentrations all along the 24 h scale.

**Figure 1** Patterns of serum cortisol in 13 patients with breast cancer. (**a**) Normal profiles ($n$=5). (**b**) Abnormal profiles ($n$=7).

**Figure 2** Patterns of serum cortisol in 20 patients with ovarian cancer. (**a**), Normal profiles ($n$=5). (**b**), Abnormal profiles ($n$=15).

*Twenty-four h profiles of CEA*

As shown in Table I, the mean values of the samples obtained at 08.00 (basal value) were found elevated in ten out of 13 breast cancer patients (range 7.6–336 ng $ml^{-1}$) and in one out of eight ovarian cancer patients (141 ng $ml^{-1}$), whereas the cut-off value is <5 ng $ml^{-1}$. Figures 3 and 4 display the individual profiles of serum CEA in breast and ovarian cancer patients respectively.

In breast cancer patients, individual profiles of CEA (Figure 3) did not follow a uniform trend and could exhibit a variability as large as 20%. No relation between the concentrations of CEA at 08.00 and cortisol pattern subgroups could be seen (Kolmogorov–Smirnov chi-square=2.223, $P=0.6581$).

The individual profiles of CEA in ovarian cancer patients are shown in Figure 4. The only patient with elevated concentration of plasma CEA had a small amplitude of variation (around 10%) and the subgroup of patients with concentrations below 5 ng $ml^{-1}$ most often showed erratic profiles. No relation between the concentrations of CEA at 08.00 and cortisol pattern subgroups could be seen (Kolmogorov–Smirnov chi-square-=1.422, $P=0.9822$).

*Twenty-four h profiles of CA 15-3 (breast cancer patients)*

As shown in Table I, the mean values of the samples obtained at 08.00 (basal value) were found elevated in any patient for CA 15-3 (range 48–490 U $ml^{-1}$), whereas the cut-off value is <25 U $ml^{-1}$.

The individual profiles of CA 15-3 also showed erratic patterns whatever the subjects overall level (Figure 5), and here again large 24 h variability was sometimes encountered. No relation between the concentrations of CA 15-3 at 08.00 and cortisol pattern subgroups could be seen (Kolmogorov-Smirnov chi-square=2.777, $P=0.4989$).

*Twenty-four h profiles of CA 125 (ovarian cancer patients)*

In 14 out of 20 ovarian cancer patients serum CA 125 concentrations at 08.00 were above the cut-off value of 35 U $ml^{-1}$ (Table I). Individual profiles of CA 125 in ovarian cancer patients are shown in Figure 6. They were arbitrarily dispatched into two subgroups, i.e. with levels below or above 100 U $ml^{-1}$. In both subgroups the serum CA 125 patterns were inconstant. In five out of 20 patients the peak concentration was observed at 08.00, whereas in three others it was their lowest one. No relation between the concentra-

**Figure 4** Twenty-four hour patterns of serum carcinoembryonic antigen in eight patients with ovarian cancer.

**Figure 3** Twenty-four hour patterns of serum carcinoembryonic antigen in 13 patients with breast cancer.

**Figure 5** Twenty-four hour patterns of serum CA15-3 in 13 patients with breast cancer.

**Figure 6** Twenty-four hour patterns of serum CA125 in 20 patients with ovarian cancer.

tions of CA 125 at 08.00 and cortisol pattern subgroups could be seen (Kolmogorov–Smirnov chi-square = 1.067, $P > 0.9999$).

### Discussion

Here we report data on the circadian patterns of serum cortisol and tumour marker antigens in 13 patients with advanced breast cancer and in 20 patients with ovarian cancer.

It is now well accepted that the circadian periodicity of cortisol secretion is an important signal for the synchronisation of human temporal structure. Independently of the distinct circadian peak coincident with the beginning of the organism's activity cycle, another low-amplitude circadian peak may be apparent in some individuals, following the midday meal and located in the early afternooon (Quigley and Yen, 1982; Follenius *et al.*, 1982). Cortisol is therefore considered as a strong oscillator, and thus as a marker of the circadian rhythmicity in man (Touitou *et al.*, 1982, 1983). Indeed, except for endocrine diseases, such as Cushing's syndrome, in which the circadian rhythm of cortisol is dramatically disrupted, and psychiatric diseases, such as depression, in which cortisol rhythm is present, although some of its parameters, e.g. the nadir and the 24 h mean are modified, cortisol rhythm is not altered by various factors, e.g. sex and aging (Touitou *et al.*, 1982, 1983). The changes in the secretory pattern of serum cortisol reported here suggest a dramatic rhythm modification: eight out of the 13 breast cancer patients (53%) had abnormal patterns of the hormone with, e.g. a flattened profile and/or shift in the peak or trough time, and/or plateau with high values in the morning. In the same way, 15 out of 20 ovarian cancer patients (75%) had abnormal secretory patterns of cortisol with either erratic peak and trough time locations and/or low concentrations of the hormone, and/or flattened profiles within the 24 h. Only five patients could be considered as having a so-called normal pattern, although two of them had low cortisol concentrations. No relationship between cortisol subgroups and concentration of tumour marker antigens in blood sampled at 08.00 could be validated with Kolmogorov–Smirnov's test. Our data thus point out the large prevalence of cortisol pattern alteration in breast and ovarian cancer patients. It has to be emphasised that considering mean group patterns results in masking the phenomenon and that pooling the data usually allows statistical validation (ANOVA, Cosinor) of a group circadian rhythm of serum cortisol (Touitou *et al.*, 1995), despite the prevalence of erratic individual profiles. The observed pattern modifications do not necessarily imply an alteration of the organism's circadian clock. Besides, the physiopathological consequences of such alterations are not yet known.

The circadian pattern of cortisol is closely related to that of adrenocorticotrophic hormone (ACTH) and other proopiomelanocortin-related hormones like $\beta$-endorphin (Gennazzani *et al.*, 1983). Recently, interleukin 1 (Dunn, 1990) and interleukin 6 (Sala *et al.*, 1990) have been added to the list of molecules dealing with ACTH secretion and action. Since an immunomodulatory circuit has been hypothesised to operate between lymphocytes and hypothalamic–pituitary–adrenal axis (Gatti *et al.*, 1994), the modifications reported here of cortisol circadian patterns may be related to changes in the immunological status of the patients. A number of other hypotheses may be raised for the origin of the alteration or masking of serum cortisol rhythm. It might be a consequence of a depressive state in cancer patients, but it is known that in depressive patients the alterations in plasma cortisol rhythm are only shifts in time of peak or trough concentrations without rhythm disruption (Kripke *et al.*, 1994). It could also be caused by a poor synchronisation of the patients related to a reduced diurnal activity and/or an alteration of sleep caused by pain and/or anxiety. Lastly, one cannot rule out a role played by the cancer itself with a possibility of erratic secretion of hormone-like substances by the tumour or its metastases.

In neither breast nor ovarian cancer patients could a relationship be found between so-called normal and abnormal serum cortisol patterns and morning concentrations of tumour marker antigens. This study allowed us to demonstrate a large variability in the 13 breast cancer patients in the individual patterns of the serum concentration of CEA and CA 15-3, two tumour marker antigens most commonly used in the follow-up of this pathology. The amplitude of these variations during the 24 h of sampling averaged 10–15%, but in some patients it could reach 30–50%. For instance, it is remarkable that the extreme values, within 24 h, of serum CA 15-3 serum concentrations could range, e.g. from 400 to 640 U $ml^{-1}$ (patient BR) and from 90 to 120 U $ml^{-1}$ (patient MP). These amplitudes were thus much greater than those observed in other patients. In the same way, extreme values for serum CEA could range from 336 to 400 ng $ml^{-1}$ in patient SF.

Antigen CA 125 is the elective tumour marker in ovarian cancer since about 80% of the patients with non-mucinous ovarian cancer have elevated blood concentrations. In the present study, 14 out of 20 ovarian cancer patients had serum concentrations of CA 125 exceeding the cut-off value of 35 U $ml^{-1}$. It has to be noted that within the 24 h the difference between the lowest and the highest concentration observed in a given subject could be as large as 70%, irrespective of the mean concentration. For example, in patients with elevated levels of the marker, serum concentrations varied from 269 to 427 U $ml^{-1}$ (subject COU) or from 175 to 300 U $ml^{-1}$ (subject NIC). This study is, to our knowledge, the first one dealing with the circadian variation of serum CA 125 in ovarian cancer and showing that, besides the modifications of the marker in non-malignant pathologies (Touitou and Bogdan, 1988), large variations of serum CA 125 can be observed within 24 h which are not related to an alteration of the clinical state of the patients, although this does not occur in the majority of cases.

Serum CEA could be documented in only eight out of the 20 ovarian cancer patients and only one had a high serum concentration of the marker. In this reduced set of ovarian cancer patients the variability of serum CEA did not exceed 10%, but it cannot be guaranteed that larger variations could not be encountered in a larger set of patients.

The abnormalities observed in this study are coherent with the hypothesis of an alteration of rhythms as already observed in other types of cancer (Focan *et al.*, 1986*a*; Touitou *et al.*, 1995; Gautherie and Gros, 1977; Klevecz *et al.*, 1987; Voutilainen, 1953). Our data on the variability of serum tumour marker antigen concentrations may express the evolutionary potential of the tumour and show that within 24 h, a 50% variability of serum concentration of a tumour marker may be only a temporary consequence of this evolution without apparent noticeable modification of the patient's clinical condition. On the other hand, marker antigens from solid tumours are stocked within the intercellular spaces. Therefore, the variability of the plasma concentration of these tumour markers as observed along the 24 h scale may reflect phenomena of a different nature from that of a circadian organisation.

In conclusion, cortisol is a marker of circadian rhythmicity allowing assessment of a subject's synchronisation, and our study shows for the first time that 22 out of 33 cancer patients have deeply altered serum cortisol circadian profiles. These data, documenting a rhythm disruption in breast and ovarian cancer patients, may be of interest in designing chronotherapy protocols since they are based upon a reliable link between clock time and the timing of the patient's functions. The rhythm alterations reported here thus raise the question of the uniformity of the time schedule of drug administration in such patients. Further studies will be necessary to establish if the resynchronisation of patients with rhythm alterations should be a prerequisite for efficient chronotherapy or would be a consequence of successful chronotherapy.

**Acknowledgements**

This work was supported by grants from the Caisse Régionale d'Assurance Maladie d'Ile de France and the Conseil Scientifique de l'Université Pierre et Marie Curie (équipe DRED EA 1538). We wish to thank Abbott laboratories (Rungis, France) and CIS biointernational (Gif-sur-Yvette, France) for their help in this study.

### References

DUNN AJ. (1990). Interleukin-1 as a stimulator of hormone secretion. *P.N.E.I.*, **3**, 26–34.

FOCAN C, FOCAN-HENRARD D, COLETTE J, MECHKOURI M, LÉVI F, HRUSHESKY WJ, TOUITOU Y AND FRANCHIMONT P. (1986*a*). Cancer-associated alteration of circadian rhythms in carcinoembryonic antigen (CEA) and alphafetoprotein (AFP) in humans. *Anticancer Res.*, **6**, 1137–1144.

FOCAN C, FOCAN-HENRARD D, FRÈRE MH, LE HUNG S, CASTRONOVO V, COLLETTE J, FRANCHIMONT P, TOUITOU Y, LÉVI F, V ROEMELING RW AND HRUSHESKY WJ. (1986*b*). Circadian CEA variability: when to sample? *J. Clin. Oncol.*, **4**, 607–608.

FOLLENIUS M, SIMON C, BRANDENBERGER G AND LENZI P. (1982). Diurnal cortisol peaks and their relation to meals. *J. Clin. Endocrinol. Metab.*, **55**, 757–761.

GATTI G, ANGELI A AND CARIGNOLA R. (1994). Chronobiology of endocrine-immune interactions. In *Biologic Rhythms in Clinical and Laboratory Medicine*, Touitou Y and Haus E. (eds) pp. 363–374. Springer Verlag: Paris.

GAUTHERIE M AND GROS C. (1977). Circadian rhythm alteration of skin temperature in breast cancer. *Chronobiologia*, **4**, 1–17.

GENNAZZANI AR, PETRAGLIA F, NAPPI C, MARTIGNONI E, DE LEO M AND FACCHINETI F. (1983). Endorphins in peripheral plasma: origin and influencing factors. In *Central and Peripheral Endorphins: Basic and Clinical Aspects*, Muller EE and Gennazzani AR. (eds) pp. 89–87. Raven Press: New York.

HAUS E AND TOUITOU Y. (1994). Chronobiology in laboratory medicine. In *Biologic Rhythms in Clinical and Laboratory Medicine*, Touitou Y and Haus E. (eds) pp. 673–708. Springer Verlag: Paris.

KLEVECZ R, SHYMKO R AND BRALY P. (1987). Circadian gating of S phase in human ovarian cancer. *Cancer Res.*, **47**, 6267–6271.

KRIPKE DF, DRENNAN MD AND ELLIOTT JA. (1994). The complex circadian pacemaker in affective disorders. In *Biologic Rhythms in Clinical and Laboratory Medicine*, Touitou Y and Haus E. (eds) pp. 265–276. Springer Verlag: Paris.

QUIGLEY ME AND YEN SSC. (1979). A mid-day surge in cortisol levels. *J. Clin. Endocrinol. Metab.*, **49**, 945–947.

REINBERG A AND SMOLENSKY M. (1983). *Biological Rhythms and Medicine. Cellular, Metabolic, Physiopathologic and Pharmacologic Aspects*, Springer Verlag: Paris.

SALAS MA, EVANS SW, LEVELL MJ AND WHICHER JT. (1990). Interleukin-6 and ACTH act synergistically to stimulate adrenal cell glands. *Clin. Exp. Immunol.*, **79**, 470–473.

TOUITOU Y AND BOGDAN A. (1988). Tumor markers in non-malignant diseases. *Eur. J. Cancer Clin. Oncol.*, **42**, 1083–1096.

TOUITOU Y AND HAUS E. (1994). *Biologic Rhythms in Clinical and Laboratory Medicine*. Springer Verlag: Paris.

TOUITOU Y, SULON J, BOGDAN A, TOUITOU C, REINBERG A, BECK H, SODOYEZ JC AND VAN CAUWENBERGE H. (1982). Adrenal circadian system in young and elderly human subjects: a comparative study. *J. Endocrinol.*, **93**, 201–210.

TOUITOU Y, SULON J, BOGDAN A, REINBERG A, SODOYEZ JC AND DEMEY-PONSART E. (1983). Adrenocortical hormones, ageing and mental condition: seasonal and circadian rhythms of plasma 18-hydroxy-11-deoxycorticosterone, total and free cortisol and urinary corticosteroids. *J. Endocrinol.*, **96**, 53–64.

TOUITOU Y, SOTHERN RB, LÉVI F, FOCAN C, BOGDAN A, AUZÉBY A, FRANCHIMONT P, VON ROEMELING RW AND HRUSHESKY WJ. (1988). Sources of predictable tumor marker variation within the so called normal range: circadian and circannual aspects of plasma carcinoembryonic antigen (CEA) in health and disease. *J. Tumor Marker Oncol.*, **3**, 351–359.

TOUITOU Y, LÉVI F, BOGDAN A, BENAVIDES M, BAILLEUL F AND MISSET JL. (1995). Rhythm alterations in patients with metastatic breast cancer and poor prognostic factors. *J. Cancer Res. Clin. Oncol.*, **121**, 181–188.

VOUTILAINEN A. (1953). Über die 24 Studen Rhythmik der Mitofrequenz in malignen Tumoren. *Acta Path. Microb. Scand.* (suppl.), **99**, 1–104.